# 16S rRNA-Based Microbiota Profiling Assists Conventional Culture Analysis of Airway Samples from Pediatric Cystic Fibrosis Patients

**DOI:** 10.1128/spectrum.04057-22

**Published:** 2023-05-18

**Authors:** Maartje Kristensen, Emma M. de Koff, Mei Ling Chu, Simone Groendijk, Gerdien A. Tramper-Stranders, Karin M. de Winter-de Groot, Hettie M. Janssens, Harm A. Tiddens, Mireille van Westreenen, Elisabeth A. M. Sanders, Bert H. G. M. Arets, Cornelis K. van der Ent, Sabine M. P. J. Prevaes, Debby Bogaert

**Affiliations:** a Department of Pediatrics, Wilhelmina Children’s Hospital, University Medical Center Utrecht, Utrecht, The Netherlands; b Spaarne Gasthuis Academy, Spaarne Gasthuis, Hoofddorp, The Netherlands; c Department of Pediatrics, Franciscus Gasthuis & Vlietland, Rotterdam, The Netherlands; d Department of Pediatric Pulmonology and Allergology, Sophia Children’s Hospital, Erasmus University Medical Center, Rotterdam, The Netherlands; e Department of Medical Microbiology and Infectious Diseases, Erasmus University Medical Center, Rotterdam, The Netherlands; f National Institute for Public Health and the Environment, Centre for Infectious Disease Control, Bilthoven, The Netherlands; g The Queen’s Medical Research Institute, University of Edinburgh, Edinburgh, United Kingdom; University of Manitoba

**Keywords:** 16S rRNA sequencing, bacterial culture, cystic fibrosis, microbiome

## Abstract

16S-based sequencing provides broader information on the respiratory microbial community than conventional culturing. However, it (often) lacks species- and strain-level information. To overcome this issue, we used 16S rRNA-based sequencing results from 246 nasopharyngeal samples obtained from 20 infants with cystic fibrosis (CF) and 43 healthy infants, which were all 0 to 6 months old, and compared them to both standard (blind) diagnostic culturing and a 16S-sequencing-informed “targeted” reculturing approach. Using routine culturing, we almost uniquely detected Moraxella catarrhalis, Staphylococcus aureus, and Haemophilus influenzae (42%, 38%, and 33% of samples, respectively). Using the targeted reculturing approach, we were able to reculture 47% of the top-5 operational taxonomical units (OTUs) in the sequencing profiles. In total, we identified 60 species from 30 genera with a median of 3 species per sample (range, 1 to 8). We also identified up to 10 species per identified genus. The success of reculturing the top-5 genera present from the sequencing profile depended on the genus. In the case of *Corynebacterium* being in the top 5, we recultured them in 79% of samples, whereas for Staphylococcus, this value was only 25%. The success of reculturing was also correlated with the relative abundance of those genera in the corresponding sequencing profile. In conclusion, revisiting samples using 16S-based sequencing profiles to guide a targeted culturing approach led to the detection of more potential pathogens per sample than conventional culturing and may therefore be useful in the identification and, consequently, treatment of bacteria considered relevant for the deterioration or exacerbation of disease in patients like those with CF.

**IMPORTANCE** Early and effective treatment of pulmonary infections in cystic fibrosis is vital to prevent chronic lung damage. Although microbial diagnostics and treatment decisions are still based on conventional culture methods, research is gradually focusing more on microbiome and metagenomic-based approaches. This study compared the results of both methods and proposed a way to combine the best of both worlds. Many species can relatively easily be recultured based on the 16S-based sequencing profile, and it provides more in-depth information about the microbial composition of a sample than that obtained through routine (blind) diagnostic culturing. Still, well-known pathogens can be missed by both routine diagnostic culture methods as well as by targeted reculture methods, sometimes even when they are highly abundant, which may be a consequence of either sample storage conditions or antibiotic treatment at the time of sampling.

## INTRODUCTION

Cystic fibrosis (CF) is a genetic disease caused by a mutation in the cystic fibrosis transmembrane conductance regulator (CFTR) gene. Despite significant advances in treatment strategies targeting this underlying defect in CF, recurrent airway infections remain an important cause of progressive lung disease. Early and optimal treatment of these infections is vital to prevent chronic inflammation and structural damage and to improve the prognosis for patients (1, 2). The choice of antibiotics to treat infections is currently culture based. However, culture-negative patients or patients colonized with multidrug-resistant bacteria are largely treated empirically, based on the experience of the physician, patients, and previous occurrence of drug allergy. Optimization of the diagnostic approach in this subgroup of patients would be preferable.

Over the past decade, sequencing techniques have become readily available. Research into the microbial community composition in CF and many other fields currently often employs culture-independent techniques. These culture-independent techniques have offered many new insights into the pathogenesis of CF-related lung infections. Where previously CF lung infections and inflammation were assumed to be caused by a few specific pathogens, we now know that generally the microbial community composition is different in CF compared with that in the healthy respiratory microbiome and that this complex ecosystem of bacteria plays a role in exacerbations of inflammation and infections (3–6). Diagnostic laboratories and clinicians still work mostly with routine culturing results for the clinical decision-making process, often focusing on the identification of a single pathogen. Many studies have looked into the application of culture-independent microbial diagnostics; however, these techniques are still far from being broadly implemented in clinical practice (7). Both culture-dependent and culture-independent techniques have advantages and disadvantages. Culture-independent techniques allow us to map the entire (bacterial) ecosystem in a specific niche and determine not only the presence but also the relative abundance of individual bacteria, as well as detect anaerobic or unculturable bacteria. When 16S rRNA-based sequencing is used, it is not always possible to taxonomically assign bacteria to the species level. Although conventional culturing is not able to detect all bacteria, it is possible to differentiate bacteria on the species level with techniques, such as matrix-assisted laser desorption ionization–time of flight mass spectrometry (MALDI-TOF MS). Another advantage of clinical culturing is the opportunity to perform antibiogram analysis. Several studies have shown recently that many more bacterial species can be cultured than had been thought previously through the use of extensive culture methods and conditions (8–10).

In this study, we aim to compare the two approaches and test whether 16S rRNA-based sequencing may assist diagnostic culturing by using a targeted reculturing approach following 16S-based microbiota profiling. In this way, the advantages of both methods (identification of a wide range of bacteria and species identification with the possibility of antibiogram analysis) could be combined in a manner feasible in clinical practice. This information might be helpful in the future to treat the previously mentioned group of “culture-negative” or therapy-resistant patients.

## RESULTS

Of all 324 original samples which were sequenced, 256 samples collected from 20 infants with CF and 43 healthy infants were still available for reculturing (see Fig. S1 in the supplemental material). After the exclusion of 10 samples which did not show any bacterial growth, 76 samples from CF children and 170 samples from healthy children were available for further analysis.

### Culture results.

M. catarrhalis was the most prevalent pathobiont (42% of samples positive) identified by routine (blind) diagnostic culturing, followed by S. aureus and H. influenzae (38% and 18% of samples positive, respectively). Less than 1% of all samples were positive for Pseudomonas aeruginosa (see Fig. S2 in the supplemental material). Gram-negative oral flora (not further specified) was identified in 48% of all samples by our primary diagnostic protocol.

Subsequently, we used selective culture plates to reculture the top-5 most abundant operational taxonomical units (OTUs) per sample based on the 16S-based sequencing profile. With this method, we detected 60 different species from 30 different genera with a median of 3 species per sample (range, 1 to 8) (see Table S2 in the supplemental material).

**FIG 1:**
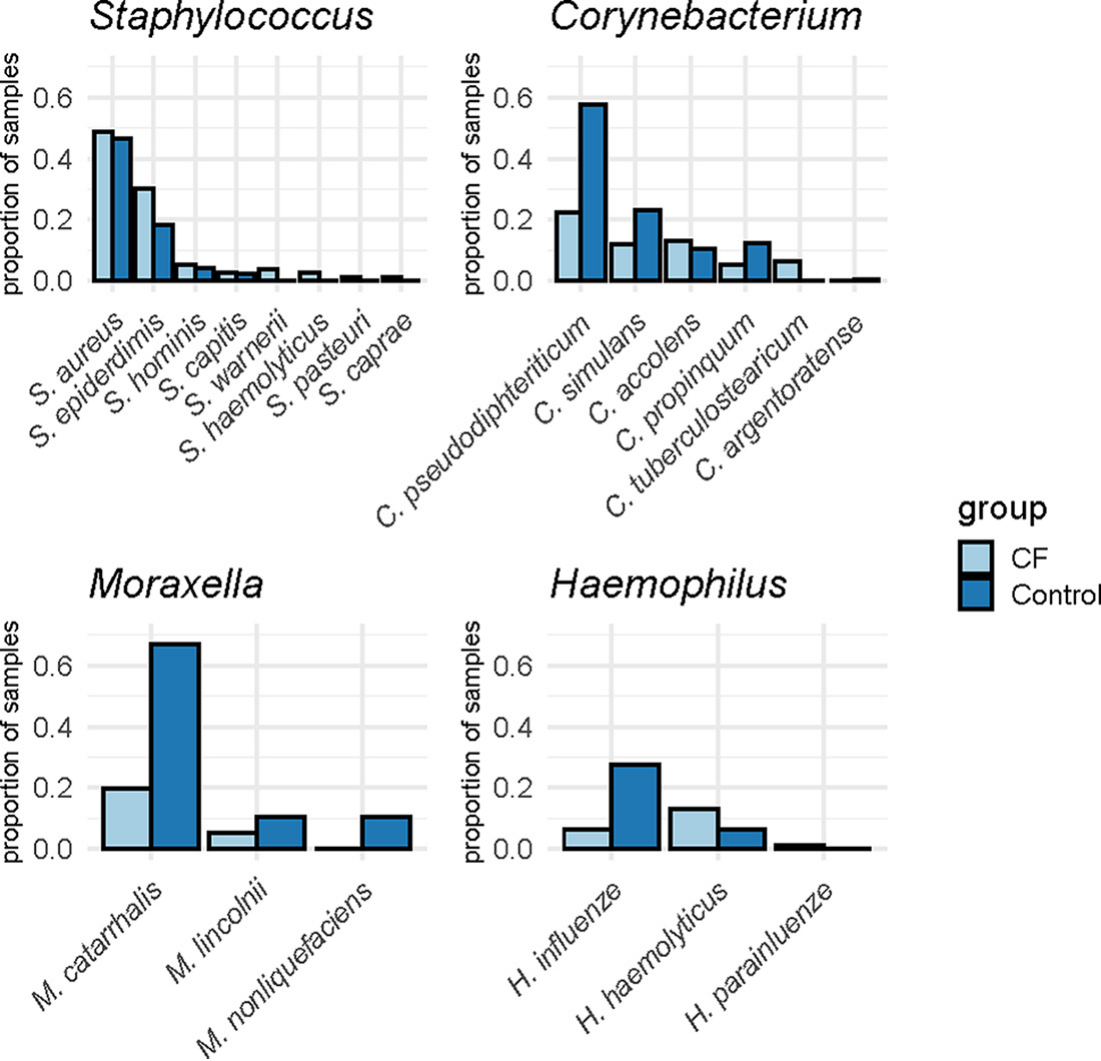
Proportion of all CF samples (*n* = 76) and control samples (*n* = 170) positive for the species representing the most abundant genera, showing most genera are represented by multiple species.

In order to compare sequence data with culture data, results from both culture approaches were aggregated; samples were considered culture positive for a specific species if they were positive with either one or both culture approaches. S. aureus was the most frequently cultured of the Staphylococcus species (47% of all samples), followed by Staphylococcus epidermidis (22% of all samples) (Fig. 1). Multiple Staphylococcus species were detected simultaneously in 12% of all the samples, with a maximum of three species per sample. The *Moraxella* genus was mostly represented by M. catarrhalis (52% of all samples) followed by Moraxella lincolnii (9% of all samples) and Moraxella nonliquefaciens (7% of all samples). Haemophilus influenzae followed by Haemophilus haemolyticus were the most abundant Haemophilus species (21% and 9% of all samples, respectively). Six different species of *Corynebacterium* were detected, of which Corynebacterium pseudodiphtheriticum was the most common (47% of all samples), followed by Corynebacterium accolens (11% of all samples). Cocarriage of different *Corynebacterium* species was detected frequently (22% of all samples), with a maximum of three different species within one sample. S. pneumoniae was the most common Streptococcus sp. and was detected in 32% of all samples.

### Culture sensitivity.

**FIG 2:**
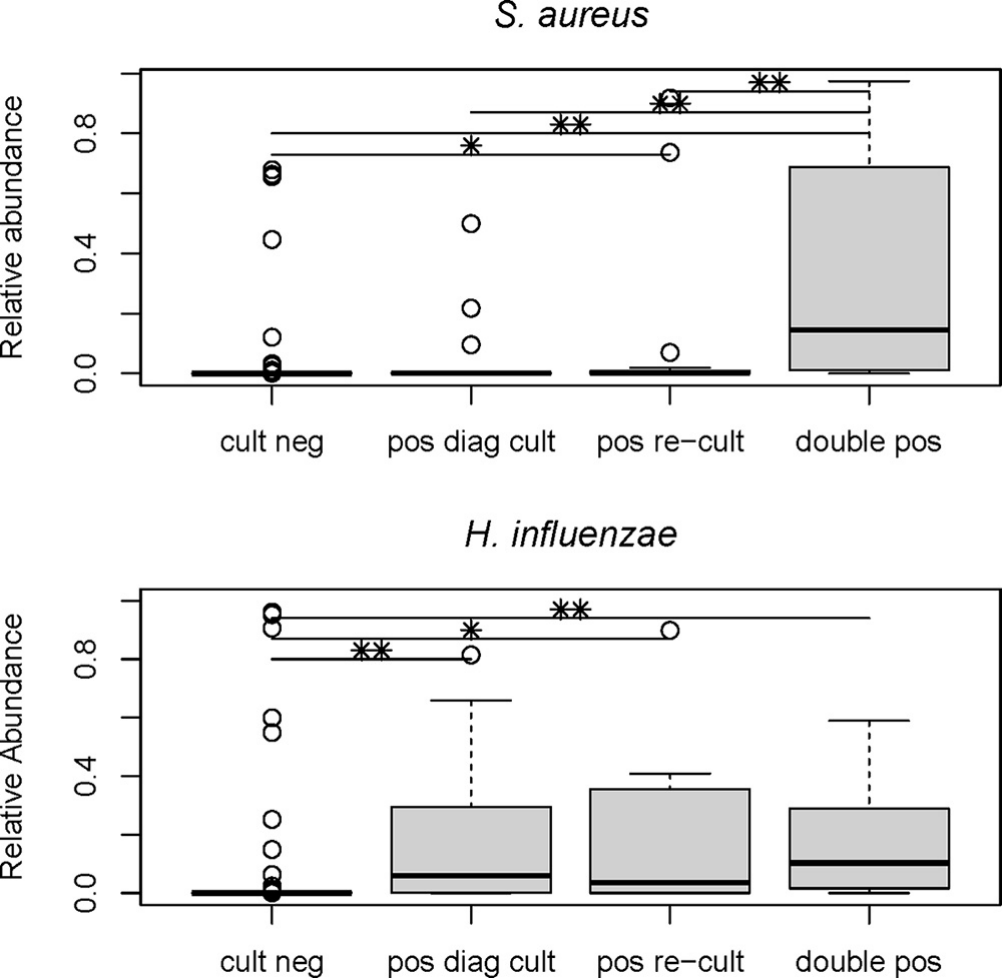
Relative abundance determined by 16S rRNA-based sequencing of S. aureus and H. influenzae for samples negative by standard diagnostic culture and reculture (cult. neg, *n* = 130 and *n* = 194, respectively), samples which were positive by either standard diagnostic culture (pos. diag. cult, *n* = 21 and *n* = 27, respectively) or by reculture (pos. recult, *n* = 23 and *n* = 7, respectively), and samples which were positive for both culture methods (double pos, *n* = 72 and *n* = 18, respectively) showing a high sensitivity of direct and delayed reculturing for S. aureus but lower sensitivity for H. influenzae. With the reculture approach, however, only samples with H. influenzae in the top-5 most abundant OTUs on Haemophilus-specific plates were used. *, *P* < 0.05; **, *P* < 0.0001.

Sequencing results from this cohort have been described previously by Prevaes et al. (4). Both S. aureus as well as H. influenzae were well identifiable up to species level from the sequencing results (4), and therefore, concordance between sequencing and diagnostic culture results could be most reliably tested for these species. We found that the relative abundances of S. aureus and H. influenzae was significantly lower in samples which were negative by diagnostic culture methods than those of culture-positive samples (*P* < 0.0001), i.e., culture becomes less sensitive when the relative abundance of a bacterium is lower (Fig. 2). Looking only at the targeted culturing results, we were able to reculture 47% of all OTUs which were selected as the top-5 most abundant species per sample. The median relative abundance of the top-5 most abundant OTUs was 4.3% (range, 0.01% to 99.7%).

There were however large differences in the success rate of reculturing between different genera. *Corynebacterium* sp. was recultured most frequently, i.e., we cultured one or more species in 140 of the 178 samples (79%) which had *Corynebacterium* as a top-5 OTU. Staphylococcus sp. was recultured least frequently, with only 19 culture-positive samples out of 77 samples (25%) with Staphylococcus as a top-5 OTU (see Table S3 in the supplemental material).

The concordance between diagnostic culture and reculture was also lower for M. catarrhalis than that for S. aureus (see Table S4 in the supplemental material). We could also identify additional strains following reculturing for pathogens generally aimed at in diagnostic culture, such as S. aureus (recultured from 23 samples [9.5%] of samples which were negative for S. aureus by diagnostic culture). M. catarrhalis was recultured from 26 samples (11%) of all samples which were negative by diagnostic culture and H. influenzae from 7 samples (2.9%) of samples negative by diagnostic culture (see Fig. S3 in the supplemental material).

### Culture and sequencing.

**FIG 3:**
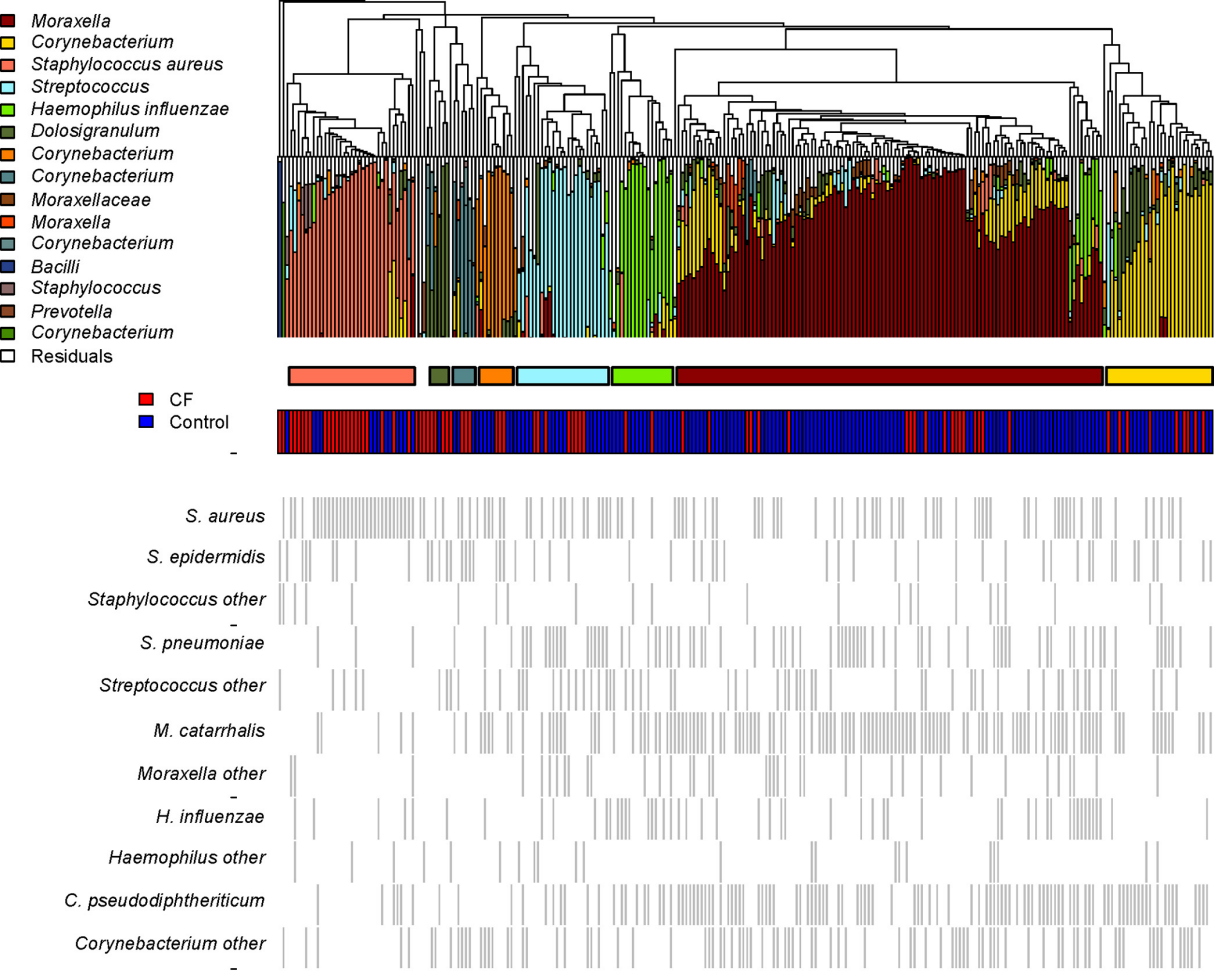
Dendrogram based on the Bray-Curtis dissimilarity of all control (blue) and CF (red) samples with the microbial composition determined by 16S rRNA-based sequencing and clusters based on the Silhouette index. The lower section (gray bars) shows the culture results of both diagnostic and reculture combined for the most frequently detected species within these same samples. For S. pneumoniae, only diagnostic culture results were taken into account, as they were confirmed by optochin testing and LytA.

A dendrogram of all samples with their microbial composition is shown in Fig. 3. When all samples were clustered based on sequencing results, 9 different clusters were distinguished. As expected, samples in the *Moraxella*-dominated cluster were most commonly culture positive for M. catarrhalis (*P* < 0.001, Wilcox signed-rank test). Culture results also show growth of S. aureus in nearly every sample in the cluster which was annotated as S. aureus. C. pseudodiphtheriticum was detected most frequently in the *Corynebacterium*-dominated cluster but also in the *Moraxella* cluster. The presence of H. influenzae and S. aureus in the sequence results was also clearly reflected in the culture data (Fig. 3).

### Differences between CF and control samples.

M. catarrhalis, C. pseudodiphtheriticum, H. influenzae, and S. pneumoniae were detected more frequently in control samples than those in CF samples, even after correction for antibiotic use, age, and repeated sampling (see Table S5 in the supplemental material). S. epidermidis was detected more frequently in CF samples; however, after correction for antibiotic use, age, and repeated sampling, this difference was not significant. There was no difference in the detection of S. aureus between control and CF samples.

## DISCUSSION

In this study, we managed to reculture many of the most abundant species from nasopharyngeal samples collected from infants with CF and healthy infants. We were able to compare the results from diagnostic culture to the 16S rRNA based sequence results and to the recultured species and study the differences between microbial culture results from infants with CF and healthy infants.

Molecular techniques are able to identify most bacteria, including the low-abundant ones, within the complex respiratory ecosystem, whereas with the methods employed for routine diagnostic culturing, only a limited number of pathogens is identified. Sibley et al. (9) already showed that by using a broad range of culture media and conditions, most respiratory bacteria can be identified with elaborate culture methods. However, in a clinical setting, these methods are not practical.

Employing a targeted approach, using 16S-based sequencing followed by reculture, we were however, able to culture many of the most abundant bacteria and identify them at species level. Importantly, we also often identified multiple species within genera in parallel. By this targeted but still relatively straightforward culture method focusing on the top 5 genera per sample only, we were able to detect 60 different species. Even for species which should initially have been detected by a (blind) diagnostic culture approach, targeted reculturing resulted in additional positive findings.

Currently, clinical antibiotic treatment for the individual patient is based frequently on physician and patient preference and previous experience. The limited sensitivity of culture which we observed in this study might in part explain the limited predictive value of results of classical culture methods on the effects of treatment with certain types of antibiotics in clinical practice (11, 12). Targeted reculturing of the most abundant species based on sequencing results might therefore better inform treatment decisions. It could give the clinician an idea of the relative abundance of the pathogens detected. Although there is still a lot unknown about the clinical implications of the relative abundance of different pathogens in CF, several studies have found associations between lower bacterial diversity and overgrowth of certain bacteria, such as *Burkholderia* spp., and clinical exacerbations and decreased pulmonary function (13–15). Additional to this information on the microbial community composition, targeted culturing could provide the clinician with more detailed information at the species level and allow for antimicrobial susceptibility testing, should it be clinically desirable. Especially in CF patients with therapy-resistant pulmonary infections and exacerbations, this method may help the clinician to make better informed decisions about the choice of antibiotic treatment.

This small study has shown that in CF microbiome research, comparing (targeted) culturing to sequencing results may further help to identify nuanced differences in colonization patterns between CF and healthy infants. For example, we observed that S. pneumoniae, H. influenzae, M. catarrhalis, and C. pseudodiphtheriticum were significantly more prevalent in healthy infants. In this cohort, Prevaes et al. (4) had already reported a higher abundance of these three species in healthy infants and a higher abundance of Streptococcus in CF. With quantitative PCR (qPCR) verification, we had already shown that S. pneumoniae is detected almost exclusively in healthy children, whereas other *Streptococci* sp., likely Streptococci mitis or Streptococci oralis are identified in CF. Prevaes et al. (4) and others (16) also found that S. aureus was significantly more abundant in CF infants. Our current reculturing data, however, show no significant differences in the prevalence of S. aureus between both groups, nuancing previous observations.

A limitation of our design was the fact that not all bacterial species identified by sequencing were present as a reference in the MALDI-TOF MS database. For example, *Dolosigranulum* sp., a common commensal in the nasopharynx of young children, was not included in the database. Moreover, distinguishing between different *Streptococcal* species with this method is difficult (17, 18). Therefore, in this case, we used LytA qPCR to confirm the presence of S. pneumoniae.

Although our diagnostic protocols are specially tailored to detect P. aeruginosa, we detected only very few positive samples in this study. In the group with CF infants, it was detected in 1 of 20 infants by the age of 6 months, which is in line with the literature on Pseudomonas detection in children of this age (19). In order to investigate the added value of a targeted culture approach on Pseudomonas detection, samples from older CF patients should be tested. The fact that all samples were cultured twice for a basic set of bacterial species, once directly after sampling with standard diagnostic culture techniques and once after storage in –80°C with more targeted reculture techniques, allowed us to evaluate the sensitivity of our culture methods for a limited number of species. We found for S. aureus a relatively high concordance between the results of both culture methods. Although many samples which contained S. aureus as a top-5 species were culture negative, the correlation between culture status and relative abundance was strong, confirming that culture has the highest sensitivity for the detection of S. aureus when it is highly abundant, although it is also detected frequently with culture from samples with a lower abundance. For M. catarrhalis, however, the concordance between both culture techniques was lower. Long-term sample storage at –80°C may have affected bacterial viability (20), although conventional culturing was done on fresh material, which also showed low sensitivity, suggesting that the identification of *Moraxella* species amid the diverse respiratory flora is challenging. A previous study also showed that the concordance between culturing and sequencing is only moderate, especially for genera like Haemophilus and Streptococcus (21). These results further emphasize that for clinical practice, one should still realize that potential pathogens may be missed by culturing, even when these pathogens are specifically targeted and sometimes highly abundant (12, 22). Therefore, having the availability of both a sequencing profile and targeted culture results might be preferable.

In conclusion, using microbial community profiles generated by 16S rRNA-based sequencing followed by a targeted reculturing approach is an efficient and reliable way to identify and isolate many of the most abundant respiratory bacteria in CF and non-CF patients. In patients with difficult or therapy-resistant infections, this method offers a broad picture of the entire bacterial community, as well as the option to obtain information, such as antibiogram analysis, of specific species and strains. This method might therefore be considered useful as a future addition to our diagnostic toolkit and deserves further exploration.

## MATERIALS AND METHODS

As described previously by Prevaes et al. (4), all infants with CF who were referred to one of two participating CF centers between 2011 and 2013 were invited to participate in our study. Healthy age and sex-matched controls were selected from the WHeezing-Illnesses-STudy-LEidsche-Rijn (WHISTLER) cohort. In short, nasopharyngeal samples from infants with CF and healthy infants were collected monthly during the first 6 months of life, using an eSwab 484CE nylon flocked flexible sterile swab (Copan Diagnostics, Brescia, Italy) and stored for transport in Amies medium. Additional samples were collected at the time of respiratory infections. All nasopharyngeal samples were cultured for Streptococcus pneumoniae, Staphylococcus aureus, Haemophilus influenzae, Moraxella catarrhalis, and Pseudomonas aeruginosa using standard diagnostic culture techniques, according to the current clinical protocol for CF samples from the upper respiratory tract and confirmed by Bruker MALDI-TOF MS. In short, samples were plated onto blood agar and Haemophilus Chocolat agar plates and incubated at 35 to 37°C for 48 h at 5% CO_2_. Additionally, all samples were cultured on MacConkey agar plates, mannitol salt agar plates, and Burkholderia cepacia selective agar 35 to 37°C under aerobic conditions for 48 h, 48 h, and 72 h, respectively. The presence of S. pneumoniae was confirmed by optochin testing and LytA qPCR because of the low distinguishing power of MALDI-TOF between different species of Streptococcus (23). Additionally, 200 μL of Amies medium containing the sample was stored with 50 μL brain heart infusion (BHI) containing 50% glycerol at −80°C, for later analysis. Subsequently, the microbial community composition of all nasopharyngeal samples collected during the study was analyzed using 16S rRNA-based sequencing (4). Following microbiota profiling, a targeted culture approach was used to reculture the most abundant bacteria, focusing on the top-5 most abundant genera per sample, using specific plates and culture conditions (see Table S1 in the supplemental material). Additionally, all samples were cultured on tryptic soy agar blood plates (BD Diagnostic Systems, Heidelberg, Germany). MALDI-TOF MS was used to provide species-level information of each unique colony type.

### Statistical analysis.

The bioinformatic pipeline which was used to analyze the sequence data has been described previously by Prevaes et al. (4). All further statistical analyses were performed in R (version 3.3.1). Cohen’s kappa was used to test the concordance between the presence for S. aureus and M. catarrhalis detected by reculturing and by diagnostic culturing. Since we cultured all samples on regular blood agar plates for the reculturing step as well as for the diagnostic culturing, these bacteria should have been detected by both methods when present. Differences between the relative abundances of culture-positive and culture-negative samples were tested using a Wilcoxon rank-sum test. A dendrogram of the sequenced samples was constructed using the Bray-Curtis dissimilarity, and the optimal number of clusters was determined by the Silhouette index (using the R package Cluster). A logistic mixed effects model was used to detect differences in bacterial presence between the CF group and the control group, after correction for antibiotic use, age, and repeated sampling (using the R-package lmerTest).

### Data availability.

The sequence data generated and analyzed during the current study will be made available in the SRA repositories, under numbers PRJNA934834 and PRJNA336315 (Table S6).
