# Supplementary material for: 16S rRNA-Based Microbiota Profiling Assists Conventional Culture Analysis of Airway Samples from Pediatric Cystic Fibrosis Patients
Source: Microbiol Spectr. 2023 May 18;11(3):e04057-22. doi: 10.1128/spectrum.04057-22 (PMC10269535; doi:10.1128/spectrum.04057-22)
Supplement: Supplemental file 1 — Supplemental material. Download spectrum.04057-22-s0001.pdf, PDF file, 0.4 MB [file spectrum.04057-22-s0001.pdf]

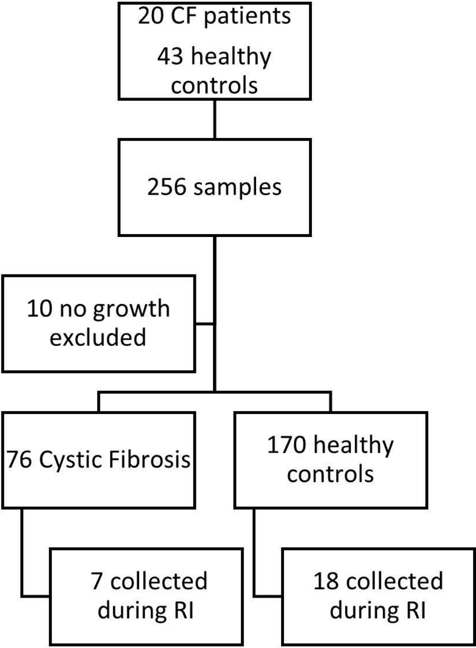
20 CF patients
43 healthy controls
256 samples
10 no growth excluded
76 Cystic Fibrosis
170 healthy controls
7 collected during RI
18 collected during RI

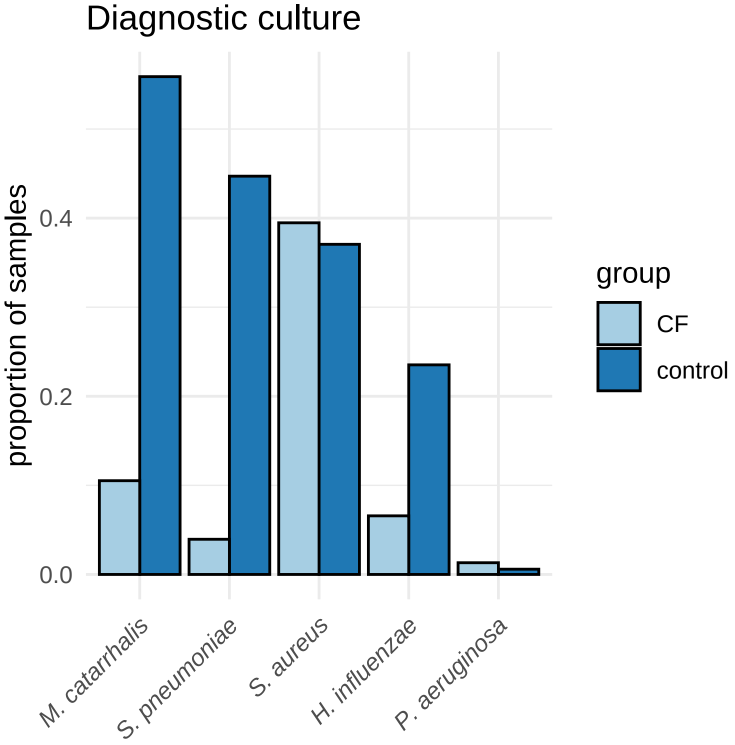

Diagnostic culture
proportion of samples
0.0
0.2
0.4
M. catarrhalis
S. pneumoniae
S. aureus
H. influenzae
P. aeruginosa
group
CF
control

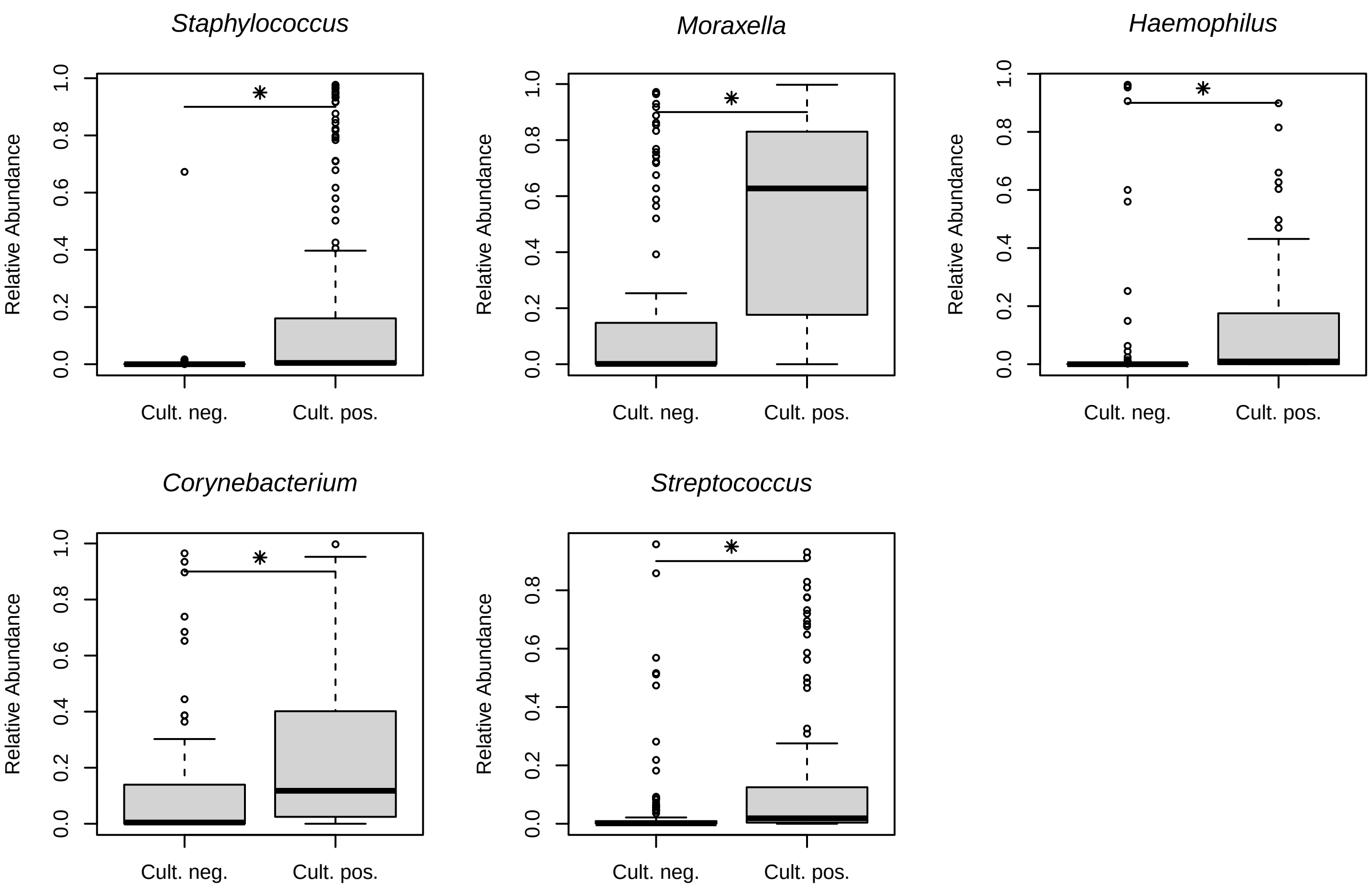

Staphylococcus
Moraxella
Haemophilus
Corynebacterium
Streptococcus
Relative Abundance
0.0
0.2
0.4
0.6
0.8
1.0
*
Cult. neg.
Cult. pos.

Supplemental materials

Figure S1: Flow-chart of characteristics of stored samples available for re-culturing from the Dutch case-control infant CF cohort. 25 of the 246 available samples were collected during a respiratory infection (RI). 76 samples were collected from infants with CF and 170 samples were collected from healthy controls.

Figure S2: Percentage of samples positive by standard diagnostic culture *M. catarrhalis, S. aureus, S. pneumoniae, H. influenzae* and *P. aeruginosa*. (Total number of samples is 246)

Figure S3: Cumulative relative abundance determined by 16S rRNA based sequencing for the most prevalent genera was significantly lower in samples in which no species of that genus were cultured compared to samples in which at least one species of that genus was cultured. * P<0.0001. Cult. pos: culture positive, cult. neg: culture negative.

**Selective culture media**	**OTU classification**
Mannitol Salt Agar<br>(BD Diagnostic Systems, Heidelberg, Germany)	*Staphylococcus, Staphylococcaceae, Dolosigranulum*
Tellurite Agar (Hoyle)<br>(BD Diagnostic Systems, Heidelberg, Germany)	*Corynebacterium*
Haemophilus II Agar<br>(bioMérieux, Craponne, France)	*Haemophilus, Pasteurellaceae*
BBL Columbia CNA Agar with 5% sheep blood<br>(BD Diagnostic Systems, Heidelberg, Germany)	*Streptococcus*
MacConkey Agar<br>(BD Diagnostic Systems, Heidelberg, Germany)	*Enterobacteriaceae, Caulobacteraceae, Comamonadaceae, Alphaproteobacteria, Bosea, Actinobacillus*
Schaedler K-V Agar<br>(BD Diagnostic Systems, Heidelberg, Germany)	*Prevotella*

**Enriched culture media**	**OTU classification**
GCII Agar with IsoVitalex<br>(BD Diagnostic Systems, Heidelberg, Germany)	*Neisseriaceae, Pasteurellaceae, Helcococcus*
Schaedler Agar with vitamin K1 and 5% sheep blood<br>(BD Diagnostic Systems, Heidelberg, Germany)	*Actinomyces, Veillonella, Fusobacterium, Porphyromonas, Lachnospiraceae*

Table S1: Selective and enriched culture media that were used to facilitate growth of species of the indicated bacterial genus, family or class represented in the top 5 most abundant operational taxonomic units (OTUs) per sample.

Species	Number of isolates	Species	Number of isolates
Staphylococcus aureus	96	*Micrococcus uteus*	4
Staphylococcus epidermidis	54	*Kocuria rhizophila*	2
Staphylococcus pasteuri	1	*Kocuria kristinae*	3
Staphylococcus hominis	11	*Gemella haemolysans*	3
Staphylococcus haemolyticus	2	*Gemella morbillorum*	4
Staphylococcus warnerii	3	*Prevotella melaninogenica*	6
Staphylococcus capitis	6	*Prevotella nanceiensis*	4
Staphylococcus caprae	1	*Actinomyces odontolyticus*	2
Corynebacterium pseudodiphtheriticum	116	*Actinomyces neuii*	1
Corynebacterium accolens	30	*Fusobacterium necrophorum*	1
Corynebacterium propinquum	27	*Solibacillus silvestris*	2
Corynebacterium tuberculostearicum	5	*Escherichia coli*	6
Corynebacterium simulans	48	*Rothia mucilaginosa*	2
Corynebacterium argentoratense	1	*Acinetobacter lwoffi*	2
Moraxella catarrhalis	81	*Dermacoccus nishinomiyaensis*	1
Moraxella nonliquefaciens	18	*Neisseria sp.*	4
Moraxella lincolnii	22	*Neisseria meningitidis*	3
*Streptococcus pneumoniae**	56	*Turicella otitidis*	1
Streptococcus mitis/oralis	42	*Solibacillus silvestris*	2
Streptococcus pyogenes	5	*Bacillus cereus*	2
Streptococcus salivarius	4	*Capnocytophaga sp.*	1
Streptococcus infantis	2	*Kingella denitrificans*	1
Streptococcus vestibularis	3	*Massilia timonae*	1
Streptococcus dysgalactiae	4	*Veilonella dispar*	2
Streptococcus parasanguinis	6	*Veilonella parvula*	2
Streptococcus agalactiae	2	*Bifidobacterium breve*	1
Streptococcus peroris	15	*Dermabacter hominis*	1
Haemophilus influenzae	25	*Eikenella corrodens*	1
Haemophilus parainfluenzae	1	*Brevibacterium casei*	1
Haemophilus haemolyticus	21	*Phorphyromonas sp.*	1

Table S2: List of isolated species from all 241 samples found with the re-culture method. **S. pneumoniae* in this table was only confirmed by MALDI-TOF MS, not by optochin testing.

	Number of times present in sample top-5	Successfully re-cultured from top-5 (number of samples, percentage)
Moraxella	170	67 (39%)
Haemophilus	59	24 (41%)
Corynebacterium	178	140 (79%)
Streptococcus	118	70 (59%)
Staphylococcus	77	19 (25%)

Table S3: Number of samples in which the selected genus is present in the top-5 most abundant taxa sequenced. From part of these samples, members of that genus could be successfully re-cultured. The number of samples from which these genera could be re-cultured, as well as the percentage of the total samples which had that genus as a top-5 taxa are given.

Species	Double negative	Pos. in diagnostic culture	Pos. in re-culture	Double positive	Cohen's Kappa
S. aureus	53%	9%	9%	29%	0.62
M. catarrhalis	48%	20%	11%	22%	0.34

Table S4: concordance calculated with Cohen's Kappa between culture results for diagnostic culture methods and re-culture methods. Percentage of samples positive for both methods, either method or neither method. For both culture methods all samples were cultured on blood agar plates, so when present, these bacteria should have been detected by both methods. However, discrepancies between detection by both methods are clearly found, suggesting limited sensitivity of culturing.

**Species**	**Number of positive control samples (%)**	**Number of positive CF samples (%)**	**Regression coefficient (95% CI)**	**P-value**
S. aureus	79 (46%)	37 (49%)	-0.43 (-1.91 – 0.93)	0.54
S. epidermidis	31 (18%)	23 (30%)	-0.60 (-1.80 – 0.59)	0.30
C. pseudodiphtheriticum	98 (58%)	17 (22%)	1.83 (0.69 – 3.25)	**<0.01**
C. accolens	18 (11%)	10 (13%)	-0.11 (-3.03 – 3.11)	0.94
Corynebacteria other	55 (32%)	18 (24%)	-0.05 (-1.57 – 1.37)	0.96
M. catarrhalis	114 (67%)	15 (15%)	2.14 (1.25 – 3.24)	**<0.001**
Moraxella other	35 (21%)	4 (5%)	1.71 (0.24 – 3.57)	**0.03**
H. influenzae	47 (28%)	5 (6.6%)	1.80 (0.44 – 3.42)	**0.01**
S. pneumoniae	76 (45%)	3 (4.0%)	4.99 (3.40 – 25.64)	**<0.01**

Table S5: Occurrence of species most prevalent in culture and difference between control and CF samples after correction for repeated sampling, age, respiratory infections and antibiotic use

Samples available from https://www.ncbi.nlm.nih.gov/sra/PRJNA336315

**Sample**	**Accession**
s1003M6	SAMN05510862
s1002M6	SAMN05510826
s1001M3	SAMN05510848
s1005M3	SAMN05510774
s1013M3	SAMN05510777
s1011M4	SAMN05510748
s1010M4	SAMN05510782
s1009M5	SAMN05510786
s1008M5	SAMN05510750
s1007M5	SAMN05510852

Table S6: All samples are available from https://www.ncbi.nlm.nih.gov/sra/PRJNA934834, except for the 10 samples listed above. These are available from https://www.ncbi.nlm.nih.gov/sra/PRJNA336315.
